# Refractive Error Stabilization Following Phacoemulsification Cataract Surgery and Its Associated Preoperative Factors

**DOI:** 10.18502/jovr.v20.15132

**Published:** 2025-09-02

**Authors:** Ali Sharifi, Davoud Dehghani-Meibodi, Amin Zand

**Affiliations:** Clinical Research Development Unit, Shafa Hospital, Kerman University of Medical Sciences, Kerman, Iran

**Keywords:** Cataract Surgery, Ocular Biometrics, Phacoemulsification, Refractive Error, Stabilization

## Abstract

**Purpose:**

To evaluate the time required for refractive error (RE) stabilization after standard phacoemulsification cataract surgery and identify preoperative factors influencing this duration.

**Methods:**

This prospective case series study enrolled patients who had undergone phacoemulsification cataract surgery. RE stabilization was defined as < 0.50 diopter changes in spherical equivalent (SE) over two consecutive follow-ups. Participants underwent ophthalmic examinations at baseline and postoperative days 3, 7, 14, 28, and 60. Keratometric values (K-mean), corneal astigmatism, and axial length (AL) were measured preoperatively using a biometric device.

**Results:**

A total of 163 eyes from 163 patients were included. RE stabilization occurred by day 28 in 98.8% of eyes, reaching 100% by day 60. Multivariate analysis revealed that age, gender, type of cataract (nuclear, cortical, or posterior subcapsular), best-corrected visual acuity, absolute SE, and K-mean did not significantly influence the time to RE stabilization (*P*s > 0.05). AL demonstrated a significant negative association with the time to RE stabilization (ß = –0.445, *P* < 0.001). Moreover, eyes with lower preoperative corneal astigmatism exhibited a shorter time to RE stabilization (ß = 1.001, *P* < 0.001).

**Conclusion:**

RE stabilization is typically observed 4 weeks post-cataract surgery. Eyes with lower AL and higher corneal astigmatism exhibit a comparatively slower RE stabilization following surgery.

##  INTRODUCTION

Following phacoemulsification cataract surgery (PCS) and intraocular lens (IOL) implantation, the stabilization of ocular refractive error (RE) is influenced by various factors. These factors encompass preoperative RE values and other ocular biometrics, surgical techniques, and the extent of postoperative corneal edema.^[1,2]^ Understanding the dynamics of RE stabilization is crucial, as any residual uncorrected RE can impact the quality of life, particularly in individuals with presbyopia.^[1,3]^ The determination of the optimal time to RE stabilization and the prescription of optical aids remain debatable in the literature. Recommendations for delaying RE correction vary across previous studies.^[2,4,5,6,7,8]^ However, a meta-analysis suggests that new glasses could be prescribed as early as one week after cataract surgery.^[9]^

Despite the importance of this period of RE stabilization, few studies have investigated the preoperative demographic and ocular factors influencing the onset of stabilization after surgery.^[2]^ Therefore, our study not only aims to assess the time required for RE stabilization following standard PCS but also explores preoperative parameters potentially associated with prolonged postoperative RE instability. For the first time, using multivariate analysis this study assesses the potential impact of several variables—including age, gender, type of cataract, best-corrected visual acuity (BCVA), absolute spherical equivalent (SE), axial length (AL), mean keratometry value (K-mean), and corneal astigmatism—on the time required for RE stabilization following cataract surgery. This dual focus addresses a gap in the current understanding of the factors influencing the dynamics of RE stabilization after cataract surgery.

##  METHODS

### Study Design 

This prospective case series study was conducted at Shafa Hospital in Kerman, Iran, from March 2022 to March 2023. The sample size was determined using the G*Power statistical software version 3.1 based on outcomes previously published by Sharifi et al.^[10]^ We selected this study because it aligns with our definition of RE stabilization.^[10]^ Assuming that RE stabilization occurred 5 weeks after PCS in 98% of eyes,^[10]^ we calculated that a minimum sample size of 138 eyes was necessary to achieve 80% power (at a 95% confidence interval) for detecting changes of < 0.50 diopters (D) in SE over two consecutive follow-ups (definition of RE stabilization). Accounting for a 20% dropout rate, we finalized the sample size at 166 eyes. To further elaborate, we used an effect size of 0.24 (calculated by the G*Power software), with an alpha value of 0.05 and a power (1- β ) of 0.8 to determine the sample size.

The study involved individuals undergoing first-eye, uncomplicated PCS with IOL implantation in the capsular bag. Patient selection was based on the presence of visually significant cataracts, defined by a Snellen BCVA of < 20/40, due to lens opacity.^[11]^ In cases of binocular visually significant cataract, the worse eye was included for analysis. Cataract categorization and grading followed the Lens Opacities Classification System III.^[12]^ We excluded participants under 50 years old with corneal astigmatism ≥ 4.00 D, AL < 20.0 mm or > 27.0 mm, mature intumescent or brunescent cataracts, anterior uveitis ≥ 2+ on postoperative day 3,^[13]^ moderate or severe corneal edema on postoperative day 3, severe comorbid eye diseases affecting visual acuity, prior ocular surgeries, combined surgical procedures, history of ocular trauma, and follow-up periods < 60 days. Demographic data and cataract characteristics were documented. This study adhered to the principles of the Declaration of Helsinki, and written consent was obtained from all participants. The study protocol was approved by the ethical committee of Kerman University of Medical Sciences, Kerman, Iran (ethical code: IR.KMU.AH.REC.1401.187).

A single examiner (DD) conducted comprehensive ophthalmic examinations at baseline and postoperative days 3, 7, 14, 28, and 60. These included RE measurement using an autorefractometer (Topcon KR-800, Topcon Corporation, Tokyo, Japan), BCVA assessments, slit-lamp biomicroscopy, Goldmann applanation tonometry to measure intraocular pressure (IOP), and dilated fundus examination using a 90-D lens. LogMAR equivalents were assigned to BCVA values for statistical analysis. Visual acuity levels as perceived by light, hand motion, and counting fingers were assigned logMAR values of 2.5, 2.3, and 1.85, respectively. RE stabilization was defined as < 0.50 D changes in SE over two consecutive follow-up visits.^[4]^

Patients underwent swept-source optical coherence tomography using the biometric device IOL Master 700 (Carl Zeiss Meditec AG, Jena, Germany) to estimate IOL power based on the Barrett Universal II formula,^[14]^ and to measure keratometry (K-mean), corneal astigmatism, and AL. A single surgeon (AS) performed all operations, employing a standard phacoemulsification technique. The incision, made with a 3.2 mm knife, was positioned temporally on the clear cornea. A one-piece hydrophobic acrylic IOL (enVista, Bausch & Lomb, Rochester, NY, USA) was implanted in the capsular bag.

### Statistical Analysis 

Statistical analysis was conducted using SPSS version 24 (IBM Inc., Chicago, IL, USA). Descriptive statistics were employed for both continuous and categorical variables, including mean, standard deviation, and range of values. The paired-samples *t*-test was utilized to evaluate differences in BCVA and corneal astigmatism between the baseline and the conclusion of the follow-up period. To assess changes over time, we performed a repeated measures ANOVA for SE values across the serial follow-ups. If a significant difference was found between means, the Bonferroni test was applied for further examination. Multiple linear regression analysis was conducted to evaluate the relationship between independent variables and the time to RE stabilization (considered as the dependent variable), as well as to assess their predictive value. Variables with a variance inflation factor exceeding 5 were deemed to exhibit excessive collinearity and were consequently excluded from the analysis. Furthermore, the independence of residual errors was assessed using the Durbin-Watson statistic. A range of 1.5 to 2.5 for this statistic is indicative of independence, and a significance level of *P* < 0.05 was considered statistically significant.

##  RESULTS

A total of 166 eyes of 166 patients were initially enrolled in the study. However, three patients were lost to follow-up before day 60 and were excluded from the analysis. Thus, the final dataset comprised 163 eyes from 163 patients, among whom 99 (60.7%) were female. The mean age of the patients was 69.00 ± 9.75 years. Preoperatively, the average BCVA was 0.91 ± 0.21 logMAR, and the baseline SE was 0.25 ± 0.42 D. Among the eyes, 90 (55.2%) exhibited nuclear sclerosis cataracts, 49 (30.1%) had cortical cataracts, and 24 (14.7%) had posterior subcapsular cataracts. The mean AL was 24.69 ± 1.76 mm. The preoperative K-mean was 44.95 ± 1.73 D, with a mean corneal astigmatism of 1.18 ± 0.84 D [Table 1].

**Figure 1:**
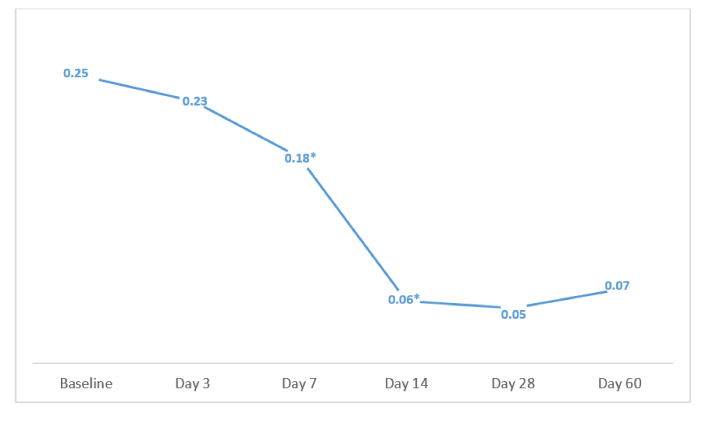
Means of spherical equivalent (SE, in diopters) during follow-up visits. Between postsurgical days 3 and 7, and also between days 7 and 14, the SE decreased significantly. For the remaining visits, the changes were not statistically significant (*P* < 0.001).

**Figure 2:**
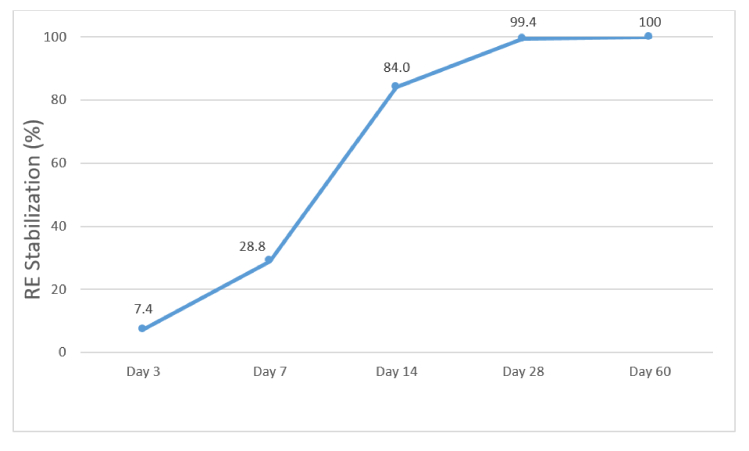
Cumulative percentage (%) of refractive error (RE) stabilization during serial follow-up visits.

At the end of follow-ups, BCVA improved to 0.11 ± 0.07 logMAR, which was statistically significant compared to the baseline (*P* < 0.001). However, at this time, corneal astigmatism was similar to the baseline value (1.17 ± 0.83 D, *P* = 1.000). On day 60, SE was 0.07 ± 0.10 D, which was a significant improvement observed during the serial follow-ups (repeated measures ANOVA, *P* < 0.001). The Bonferroni test demonstrated a substantial reduction at all follow-up assessments relative to baseline. Notably, there was a significant decrease on day 7 compared to day 3, and on day 14 compared to day 7 (*P*s < 0.001) [Figure 1]. However, RE stabilization (defined as < 0.50 D changes in SE over two consecutive follow-up visits) occurred in nearly all cases by day 28. Specifically, it occurred by day 14 in 84.0% of eyes, reaching 99.4% by day 28 and 100% by day 60 [Figure 2].

In the univariate analysis, only corneal astigmatism was significantly associated with the time to RE stabilization (*P* < 0.001). No significant associations were observed between the time to RE stabilization and other variables such as age (*P* = 0.372), gender (*P* = 0.309), cataract type (*P* = 0.181), BCVA (*P* = 0.528), absolute SE (*P* = 0.218), AL (*P* = 0.078), or K-mean (*P* = 0.884). However, after multivariate analysis, both AL and corneal astigmatism were found to be significantly associated with the time to RE stabilization. AL showed a significant negative association with the time to RE stabilization (ß = –0.445, *P* < 0.001), while lower preoperative corneal astigmatism was linked to a shorter time to RE stabilization (ß = 1.001, *P* < 0.001) [Table 2].

**Table 1:** Baseline demographics and ocular characteristics

Variable	Total number (*n*) = 163 eyes of 163 patients
Age (yrs), mean ± SD (range)	69.00 ± 9.75 (50 to 94)
Gender, *n* (%)	Female: 99 (60.7) Male: 64 (39.3)
Type of cataract, *n* (%)	Nuclear sclerosis: 90 (55.2) Cortical: 49 (30.1) Posterior subcapsular: 24 (14.7)
BCVA (logMAR), mean ± SD (range)	0.91 ± 0.21 (0.41 to1.41)
SE (D), mean ± SD (range)	0.25 ± 0.42 (–0.91 to 1.38)
AL (mm), mean ± SD (range)	24.69 ± 1.76 (20.37 to 26.99)
K-mean (D), mean ± SD (range)	44.95 ± 1.73 (40.7 to 50.5)
Corneal astigmatism (D), mean ± SD (range)	1.18 ± 0.84 (0.0 to 3.5)
BCVA, best-corrected visual acuity; logMAR, logarithm of the minimal angle of resolution; SE, spherical equivalent; K-mean, mean keratometry; AL, axial length; D, diopter; SD, standard deviation, yrs, years; n, number.	

**Table 2:** Univariate and multivariate regression analyses for evaluation of the impact of preoperative demographic and biometric factors on the time of postoperative RE stabilization

**Variable**	**Univariate analysis**			**Multivariate analysis**		
	**Standard partial regression coefficient (ß)**	**95% CI **	***P*** **-value**	**Standard partial regression coefficient (ß)**	**95% CI**	***P*** **-value**
Age	–0.070	–0.183, 0.069	0.372	–0.011	–0.041, 0.023	0.567
Gender	–0.080	–3.809, 1.213	0.309	–0.024	–1.013, 0.246	0.231
Type of cataract	–0.105	–2.809, 0.534	0.181	–0.022	–0.666, 0.194	0.280
BCVA (logMAR)	0.050	–4.039, 7.845	0.528	0.012	–1.606, 2.540	0.657
Absolute SE	0.097	–1.591, 6.911	0.218	0.004	–1.994, 2.230	0.912
AL	–0.139	–1.973, 0.105	0.078	–0.445	–3.390, –2.604	< 0.001
K-mean	0.012	–0.662, 0.767	0.884	0.031	–0.035, 0.320	0.114
Corneal astigmatism	0.873	7.526, 8.960	< 0.001	1.001	9.049, 9.861	< 0.001
BCVA, best-corrected visual acuity; logMAR, logarithm of the minimal angle of resolution; SE, spherical equivalent; K-mean, mean keratometry; AL, axial length; CI, confidence interval.						

##  DISCUSSION

The period of RE instability following PCS is critical, as uncorrected RE during this phase may negatively impact patients' quality of life—particularly in presbyopic individuals who depend on near-vision glasses for daily tasks, even during this transient period before RE stabilization.^[1,3]^ Therefore, identifying factors that predict the onset of RE stabilization is essential for estimating postoperative RE correction timelines.

In our study, using multivariate analysis, we investigated the influence of various demographic and ocular characteristics on the time to RE stabilization over a 2-month follow-up period. The findings contribute to the understanding of factors affecting the postoperative stability of RE.

The timeframe for RE stabilization after standard PCS varies across previous studies.^[2,4,5,6,7,8][10]^ Ostri et al^[5]^ found automated refraction could stabilize 1 week post-surgery, while Berk et al^[4]^ reported 78.8% of operated eyes achieved RE stabilization by week 3. Sugar et al^[6]^ demonstrated stable SE (within +/–0.50 D of the 4-month refraction) in 66.1% of cases at week 1 and 78.1% at month 1. De Juan et al^[7]^ showed stability in both spherical and cylindrical refraction 1 week post-surgery. McNamara et al^[8]^ observed RE stabilization in 59% and 75% of cases at weeks 2 and 4, respectively. Charlesworth et al,^[9]^ in a systematic review and meta-analysis, suggested providing new glasses 1 week after surgery. In their meta-analysis, no statistical difference was detected when comparing sphere, cylindrical, and SE values at 1 week and 4 weeks postoperatively. However, they showed that cylindrical instability may be detected in some patients as early as 1 week after surgery. In our study, RE stabilization was observed in 84.0% of eyes at 2 weeks and in 99.4% at 4 weeks post-surgery. Some of the reviewed studies evaluated the time to RE stabilization based on statistical differences in SE across serial postoperative follow-up periods.^[5,6,7]^ However, similar to the study by Berk et al,^[4]^ we adopted the definition of < 0.5 D changes in absolute SE for the stabilization of postoperative RE. Despite the potential limitations and biases associated with these two statistical and clinical methods for evaluating the time to postoperative RE stabilization, previous studies have consistently demonstrated statistically insignificant changes in RE, typically within a range of ± 0.5 D.^[6]^ Additionally, the literature suggests a correlation between larger corneal incisions and delayed postoperative stabilization.^[15,16,17]^ Therefore, the time to RE stabilization may decrease in modified PCS operations, including small incision techniques and femtosecond laser-assisted cataract surgery (FLACS). Khan et al^[1]^ proposed that RE could stabilize within 1 week after small incision PCS. Similarly, Conrad-Hengerer et al18] observed RE stabilization at week 1 after FLACS, but not until month 1 after standard PCS with a 2.75 mm corneal incision. Conversely, Berk et al^[4]^ found no significant difference in the time to RE stabilization between eyes undergoing FLACS and those undergoing standard PCS with a 2.2 mm corneal incision; both groups reached RE stabilization by week 1 postoperatively. However, our cases were operated on with a 3.2 mm corneal incision, which may have contributed to a longer time to postoperative RE stabilization (4 weeks) compared to the study by Berk et al.^[4]^ Furthermore, other factors such as central corneal thickness, type of implanted IOL, capsular bag shrinkage, and changes in IOL position should be considered when assessing differences in the time to postoperative RE stabilization.^[19,20,21,22]^ In summary, disparities between our findings and those of previous studies may arise from variations in the definitions of RE stabilization, differences in surgical procedures, and variations in participant and ocular characteristics.

Consistent with our results, Khan et al^[1]^ reported no noteworthy differences based on age or gender in the duration required for RE stabilization following cataract surgery. Nevertheless, other studies have indicated that elderly individuals, particularly females, might encounter diminished visual functions during short-term follow-ups after cataract surgery.^[9,23,24]^

Previous studies have demonstrated that the time to RE stabilization can be influenced by ocular structural and biometrical factors, possibly resulting in shorter stabilization periods in emmetropic eyes.^[25]^ Mrugacz et al^[2]^ revealed RE stabilization after cataract surgery occurred in the third week in 91% of emmetropic, 77% of myopic, and 46% of hypermetropic eyes. Patients with axial myopia, characterized by more relaxed lens zonules, larger vitreous cavities, and increased vitreous liquefaction, may experience an extended time to RE stabilization after cataract surgery and IOL implantation. On the other hand, eyes with axial hypermetropia and a smaller lens capsular diameter may undergo early bending of the optic-haptic junction of the IOL, which could normalize over time.^[2]^ Our findings revealed a significant delay in the time to RE stabilization in eyes characterized by lower AL.

In a review, Freitas et al^[26]^ showed that 64.6% of eyes that were candidates for cataract surgery had corneal astigmatism between 0.25 and 1.25 D, and 22.2% had astigmatism of ≥ 1.50 D. In another study by De Juan et al,^[7]^ the baseline cylindrical refraction in eyes that were candidates for standard PCS was 1.39 ± 0.94 D, which stabilized 1 week after the surgery. In our study, the value of baseline corneal astigmatism was comparable to that in these studies (1.18 ± 0.84 D). According to our observations, in eyes with lower baseline corneal astigmatism, the RE stabilized earlier compared to those with higher values.

The strengths of our study include its prospective design and comprehensive assessment of preoperative factors. However, we had some limitations. First, we did not separately evaluate changes in spherical and cylindrical refractions during follow-ups, using SE as a combined variable. Second, we did not measure subjective refraction as the gold standard of refraction measurement. However, previous studies have reported good reliability for objective refractions, including autorefractor measurements, when compared with the subjective measurement methods in pseudophakic eyes.^[27]^ Third, although we noted a significant negative association between AL and the time to RE stabilization, we only included eyes with AL between 20.0 and 27.0 mm. Therefore, this association may not exist when high hyperopic eyes and/or high myopic eyes are included in the sample. Fourth, certain pre- and intraoperative factors, such as central corneal thickness, the axis of corneal astigmatism, as well as phacoemulsification time and energy, were not evaluated in the study. Furthermore, we did not evaluate the participants for dry eye disease, which may affect the RE measurements.^[28]^

In summary, our study indicates that RE stabilization typically occurs approximately 4 weeks after standard PCS for the majority of cases. Eyes exhibiting lower AL and increased corneal astigmatism notably demonstrate a prolonged period for RE stabilization. Recognizing these variables can enhance the development of personalized postoperative care plans, thereby optimizing outcomes for individuals undergoing cataract surgery.

##  Financial Support and Sponsorship

None.

##  Conflicts of Interest

None.
